# Foetal Alcohol Spectrum Disorder: Under-Recognised and Missed: A CAMHS Case Series Exploring Neurocognitive Profiles

**DOI:** 10.1192/bjo.2026.11894

**Published:** 2026-06-30

**Authors:** Rani Samuel, Amanda Waldman, Ruth Webster, Holly Dawson

**Affiliations:** 1 Kings Maudsley Partnership, London, United Kingdom; 2 Kings College London, London, United Kingdom

## Abstract

**Aims:**

Background:

Foetal Alcohol Spectrum Disorder (FASD) is under-recognised in Children in Care (CiC), Adopted Children (AC) and those on Special Guardianship Order (SGO) known to Child and Adolescent Mental Health Services (CAMHS. While neurodivergent conditions such as Attention Deficit Hyperactivity Disorder (ADHD), Autism Spectrum Disorder (ASD), or behavioural problems are recognised, underlying neurocognitive impairments associated with Prenatal Alcohol Exposure (PAE) remain rarely explored. This case series aims to describe the neurocognitive profiles of children diagnosed with FASD identified via local diagnostic pathways, in the CiC specialist CAMHS ‘Symbol’ team.

**Methods:**

Case Report:

Of the four children reviewed on the neurodevelopmental care pathway, three received a multidisciplinary diagnosis of FASD. Assessments included detailed mental health assessment, developmental and family histories, school reports and information collated from CiC medical records, OT (Occupational Therapy), SALT (Speech and Language Therapy), EP (Educational Psychology) reports and social care records. Standardised cognitive assessments (WISC-V -including full-scale IQ and domain-level profiles) and Adaptive Behaviour Assessment System (ABAS) were completed. Particular attention was given to variability across cognitive domains, adaptive functioning, and discrepancies between verbal ability, working memory, processing speed and fluid reasoning. The Scottish SIGN guidance was used to guide diagnostic process.

**Results:**

Discussion:

There was variability in overall cognitive functioning across the children assessed, ranging from Mild Learning Disability to Low Average cognitive ranges. Across cognitive domains, there was great variability, with the majority showing relative strengths in Verbal Comprehension. There was variability in the Working Memory domain across assessments, and all children showed significant relative impairments in Processing Speed, Fluid Reasoning and Visuo-Spatial domains. This “spiky profile” contributed to diagnostic overshadowing, as average/ low average full-scale IQ scores sometimes masked clinically significant cognitive impairments at domain level. The ABAS often revealed gaps between cognitive abilities and real-world functioning.

**Conclusion:**

Conclusion:

FASD may be under-diagnosed in CAMHS populations, particularly where children present with complex needs and uneven cognitive profiles. Awareness of “spiky” neurocognitive patterns and systematic multidisciplinary assessment can improve diagnostic accuracy and inform educational and therapeutic support. The ABAS, alongside cognitive assessments are important in identifying domains of difficulty in the real world. Early identification has significant implications for care-planning, family understanding and psychological support in receiving a diagnosis of FASD.

